# Prosthetic Status, Removable Prostheses and Quality of Life in Older Adults: A Retrospective Cross-Sectional Analysis Within a Population-Based Study

**DOI:** 10.3390/dj14010007

**Published:** 2025-12-22

**Authors:** Kinga Wnorowska, Katarzyna Dębkowska, Zuzanna Borawska, Stefanie Samietz, Joanna Bagińska, Inga Kamińska, Marlena Dubatówka, Zofia Stachurska, Paweł Sowa, Karol A. Kamiński, Magdalena Nowosielska

**Affiliations:** 1 Dental Surgery “Dental Plus Stomatologia”, 02-237 Warsaw, Poland; 2 Faculty of Economics and Finance, University of Białystok, 15-062 Białystok, Poland; 3 University Hospitals Coventry and Warwickshire Trust, Coventry CV2 2DX, UK; 4 Department of Prosthodontics, Gerodontology and Dental Materials, University Medicine Greifswald, 17475 Greifswald, Germany; 5 Department of Dentistry Propaedeutics, Medical University of Białystok, 15-295 Białystok, Poland; 6 Department of Integrated Dentistry, Medical University of Białystok, 15-276 Białystok, Poland; 7 Department of Population Medicine and Lifestyle Diseases Prevention, Medical University of Białystok, 15-269 Bialystok, Poland; 8 Department of Gerostomatology, Medical University of Białystok, 15-267 Białystok, Poland

**Keywords:** tooth loss, partial denture, dentures, quality of life, aged, cross-sectional studies

## Abstract

**Background/Objectives:** Tooth loss is a key marker of ageing and is linked to functional, psychological, and nutritional decline. Removable dental prostheses (RDPs) are widely used, yet their impact on life satisfaction and oral health–related quality of life (OHRQoL) remains uncertain. This study aimed to: (1) identify dental deficiencies in adults aged ≥50 years; (2) assess the use of RDPs; and (3) examine the relationship between prosthetic status, life satisfaction, and OHRQoL. **Methods:** This retrospective cross-sectional analysis included 986 participants from the Białystok PLUS cohort (2018–2024). Dental examinations classified individuals into: Group 0 (no deficiencies), Group 1 (deficiencies without prostheses), and Group 2 (deficiencies with RDPs). Life satisfaction was measured using the Satisfaction with Life Scale (SWLS), and OHRQoL using the Geriatric Oral Health Assessment Index (GOHAI). Analyses included Kruskal–Wallis test and correspondence analysis. **Results:** Partial mandibular deficiencies were the most frequent and were less often rehabilitated than maxillary defects. Most prostheses were mucosa-supported. Life satisfaction (mean SWLS = 22.4) did not differ significantly between groups (*p* = 0.326). In contrast, OHRQoL differed significantly (mean GOHAI = 53.8; *p* = 0.0001), supporting an effect of prosthetic status. Group 0 showed the highest GOHAI scores (55.7), while Group 2 (52.7) scored lower than Group 1 (53.0). Prosthesis users most often reported dissatisfaction with appearance and eating comfort. **Conclusions:** Life satisfaction appears independent of prosthetic status. OHRQoL, however, is strongly associated with dentition: individuals without deficiencies report the highest outcomes, whereas RDPs—especially mucosa-supported types—do not improve, and may reduce, perceived OHRQoL.

## 1. Introduction

Numerous studies have demonstrated a strong association between accelerated ageing processes and oral diseases [1,2]. Inflammatory, viral, and neoplastic conditions affecting the periodontium, oral mucosa, and dental tissues frequently lead to tooth loss and impaired masticatory function. These consequences have been linked to cognitive decline [3,4,5], reduced functional independence [6,7,8], psychological distress [9], and deterioration in dietary quality [10,11], all of which may diminish overall well-being and quality of life [12].

Tooth loss and edentulism—the most severe outcomes of oral disease—are recognised as important indicators of biological ageing [12,13,14]. In the Global Strategy on Oral Health 2023–2030, the World Health Organisation (WHO) emphasises the importance of preserving natural dentition and integrating oral health into universal health coverage [15]. According to the Global Burden of Disease 2015 study, nearly half of the world’s population experienced disability from oral disorders, with complete tooth loss remaining one of the leading contributors to disability-adjusted life years (DALYs) [14].

Oral rehabilitation with dental prostheses remains the main approach for restoring mastication, speech, and aesthetics. Increasingly, treatment outcomes are evaluated using oral health–related quality of life (OHRQoL) measures [16,17]. However, removable dental prostheses (RDPs) have shown variable patient acceptance, particularly concerning chewing efficiency, dietary limitations, aesthetics, and social functioning [18,19]. Several studies indicate that quality of life among those with tooth loss may remain similarly compromised regardless of prosthesis use [20,21], and in some cases, RDPs may fail to improve—or may even worsen—self-reported comfort and well-being [19].

### Life Satisfaction vs. OHRQoL: Conceptual Distinction and Relevance

A major conceptual challenge in geriatric health research is differentiating life satisfaction, a global and stable cognitive judgment about one’s life as a whole, from OHRQoL, which reflects the functional, psychosocial, and symptomatic impacts of oral conditions. Life satisfaction is shaped by broader psychological, social, and cultural factors and often remains stable even when objective health declines. In contrast, OHRQoL is more sensitive to specific oral impairments, including tooth loss, masticatory limitations, pain, and the presence or absence of prostheses.

Emphasising this distinction is a particular strength of our study, as few population-based analyses evaluate both constructs simultaneously. Evaluating life satisfaction alongside OHRQoL allows for identification of subtle discrepancies such as the “satisfaction paradox”, in which individuals maintain high global life satisfaction despite significant oral or systemic health problems. The combined use of these measures provides a more nuanced profile of well-being and supports more person-centred interpretation of clinical and prosthetic outcomes [22,23].

Recent global perspectives on healthy ageing stress the importance of maintaining functional capacity that supports well-being in older adulthood [24]. Validated, culturally adapted instruments play a critical role in capturing these subjective outcomes.

In this context, we conducted a retrospective population-based cross-sectional study with three primary objectives: (1) to describe the prevalence and types of dental deficiencies in a representative urban population aged ≥50 years; (2) to assess the types of RDPs used; and (3) to evaluate how prosthetic status relates to both OHRQoL and life satisfaction. Based on these aims, the following null hypotheses (H_0_) were formulated: (1) prosthetic status has no effect on OHRQoL; (2) prosthetic status has no effect on life satisfaction; and (3) among individuals with dental deficiencies, the use of RDPs does not improve either outcome. These hypotheses correspond to comparisons between participants without deficiencies, those with unreconstructed deficiencies, and those using removable restorations.

## 2. Materials and Methods

### 2.1. Study Population

This retrospective cross-sectional study used data from residents of Białystok (the largest city in north-eastern Poland) collected within the Białystok PLUS cohort (Poland’s largest long-term study of the health status of the urban population) [25]. Participants were randomly selected from the city registry to reflect the demographic structure of residents aged 20–80 years at the time of recruitment. Our analysis focused on individuals enrolled between November 2018 and June 2024. The study period (November 2018–June 2024) corresponded to the examination period of the first Białystok PLUS cohort.

Of the 7294 invited individuals, 2744 reported for examination. Dental examinations were conducted by four calibrated dentists under identical conditions (artificial light, no suction or air stream). Inter-examiner reliability among the four calibrated dentists was assessed using Cohen’s kappa, with values ranging from 0.91 to 1.00, indicating very high to almost perfect agreement.

This study was approved by the Ethics Committee of the Medical University of Białystok (approval nos. R-I-002/108/2016; APK.002.148.2024, dates: 31 March 2016 and 22 February 2024, respectively) and conducted in accordance with the Declaration of Helsinki. All participants provided written informed consent.

### 2.2. Sample Selection

#### 2.2.1. Age

Tooth loss is a widely used epidemiological marker of oral health, and its risk varies with age and comorbid conditions such as caries and periodontal disease [14,26]. Evidence shows that early tooth loss (<45 years) increases the likelihood of further tooth loss in later life, while after age 55, the risk rises sharply each year [14]. The Global Burden of Disease 2015 study also highlighted the sixth decade of life as a critical point when tooth loss risk increases, particularly due to periodontal causes [14].

Based on this evidence, we included participants aged ≥50 years. They were stratified into three age groups: 50–59 years, 60–69 years, and 70–82 years. Age group 70–82 years included 2 subjects who had turned 80 between the recruitment and the examination. The final study sample comprised 986 participants over 50 years of age. Considering the 2018 Białystok population aged 50–80 years (91,902 residents), this sample size corresponds to a 3% estimation error at a 95% confidence level.

#### 2.2.2. Dental Data

Prosthetic status was assessed separately for the maxilla and mandible. Deficiencies were classified as partial (teeth remaining in the arch) or complete (edentulous arch). Participants were then categorised into three prosthetic groups dependent on RDP use (Table 1). This three-category classification was developed specifically for the purposes of this study, as no existing system adequately captured the distinctions required for analysing prosthetic status in this population.

#### 2.2.3. Life Satisfaction and Quality of Life

Life satisfaction was measured using the Satisfaction with Life Scale (SWLS) [27]. This five-item instrument uses a 7-point Likert scale (1 = strongly disagree, 7 = strongly agree). Total scores (range 5–35) were categorised as: 31–35 (extremely satisfied), 26–30 (satisfied), 21–25 (slightly satisfied), 20 (neutral), 15–19 (slightly dissatisfied), 10–14 (dissatisfied), 5–9 (extremely dissatisfied).

OHRQoL was assessed with the Geriatric Oral Health Assessment Index (GOHAI) [28], which contains 12 items across four dimensions: functional limitation (items 2–4), pain/discomfort (items 5, 8, 12), psychological impact (items 7, 9–11), and behavioural impact (items 1, 6). Each item is scored on a 5-point Likert scale. Items 3, 5, and 7 are reverse-coded. Scores range from 12 to 60, with higher values indicating better oral health–related quality of life: 57–60 = very good, 51–56 = moderate, <50 = poor. Because the original questionnaires were in English, they were translated, culturally adapted, and validated in Polish through pilot studies [29,30]. Participants completed questionnaires independently, and both dental and questionnaire assessments were conducted on the same day. Cronbach’s alpha was calculated to assess the internal consistency of each questionnaire in the total sample (values reported in the Results); the coefficient was not used for between-group comparisons.

**Figure 1:**
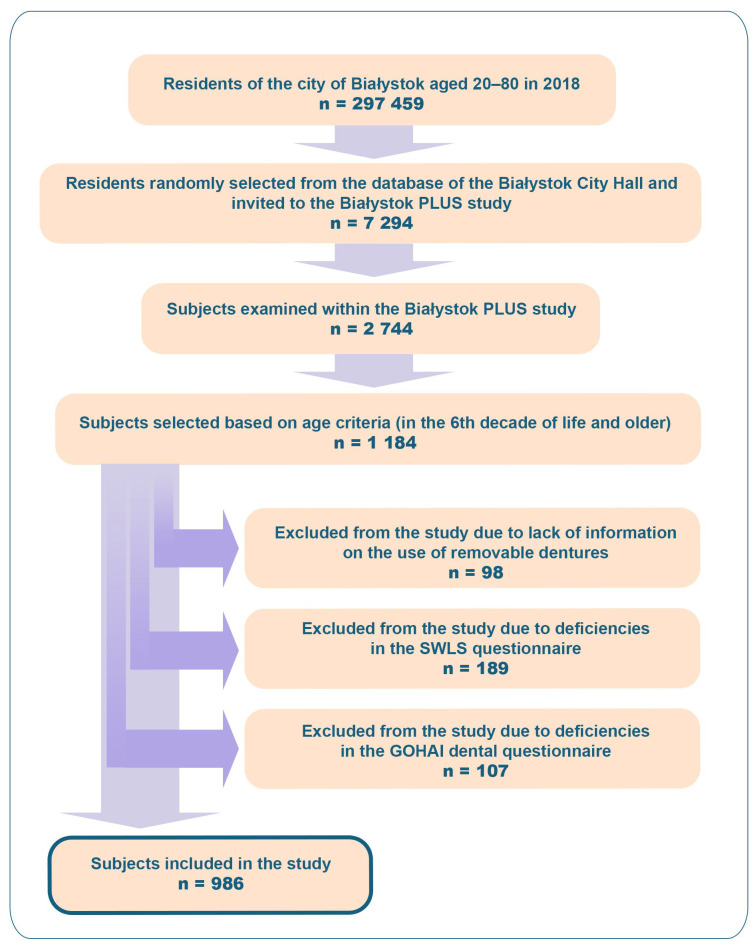
Flow chart of sample selection.

Exclusion criteria were: (1) incomplete dental examination preventing classification of deficiencies/prosthetic status, (2) incomplete SWLS responses, and (3) missing GOHAI responses. Sample selection is shown in Figure 1. Some subjects met more than one exclusion criteria. This study adhered to Strengthening the Reporting of Observational Studies in Epidemiology (STROBE) guidelines [31].

### 2.3. Statistical Analysis

To test the hypotheses, we used descriptive and inferential statistics. Normality of SWLS and GOHAI distributions was assessed in preliminary analyses and both scales showed deviations from normality; therefore, group comparisons were conducted using the non-parametric Kruskal–Wallis test [32,33]. Because both instruments also allow for categorical interpretation, correspondence analysis was applied to visualise associations between prosthetic status groups and the categorical levels of SWLS and GOHAI in a two-dimensional space [34,35].

## 3. Results

The final study sample included 986 participants: 563 women and 423 men. The largest subgroup comprised individuals aged 60–69 years and retired participants (Table 2). Age, sex, and labour market participation were reported descriptively to characterise the study population; however, inferential analyses focused exclusively on comparisons between prosthetic status groups, in line with the study objectives.

### 3.1. Prosthetic Status

Partial dental deficiencies were common, particularly in the mandible, where they occurred more frequently and were less often rehabilitated than comparable maxillary defects. When prostheses were used, they were predominantly mucosa-supported; tooth- and implant-supported options accounted for only a small proportion of restorations. Unreconstructed partial deficiencies were widespread, especially in the mandible, whereas complete edentulism—though less prevalent—was almost always rehabilitated. These overall patterns are summarised in Table 3.

Demographic characteristics (age, socioeconomic status, and labour market position) are shown in Table 2. Men were more likely to present with unreconstructed deficiencies, while women were more often represented among prosthesis users. Younger participants were concentrated in Group 0 (no deficiencies), while older individuals were more evenly distributed across Groups 1 and 2. The oldest age category was particularly represented among prosthesis users, consistent with cumulative tooth loss associated with ageing. Labour market participation also varied across groups: employed individuals—typically younger and healthier—were most common in Group 0, whereas retirees formed the largest proportion of prosthesis users in Group 2. Unemployed individuals and disability pensioners were more often found in Group 1, reflecting a pattern in which those with limited economic resources were less likely to receive prosthetic rehabilitation.

### 3.2. Prosthetic Status and Life Satisfaction

**Figure 2:**
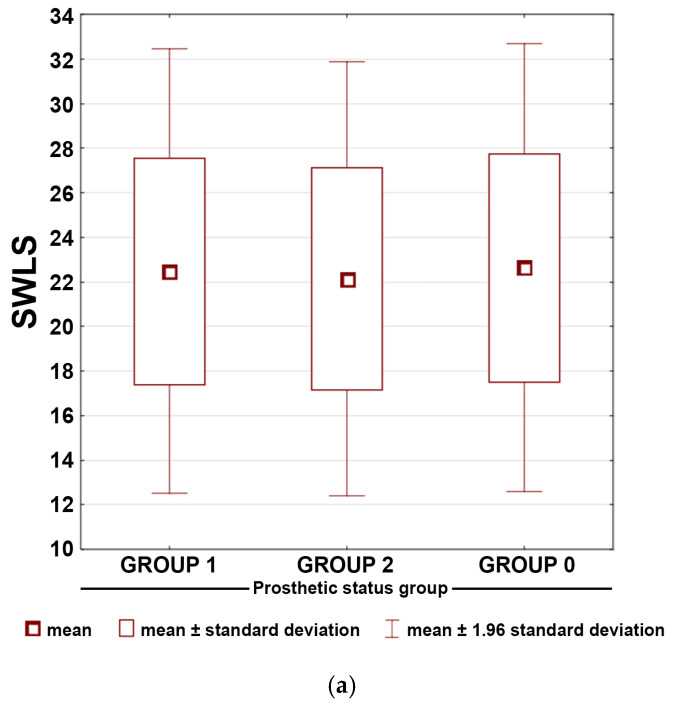

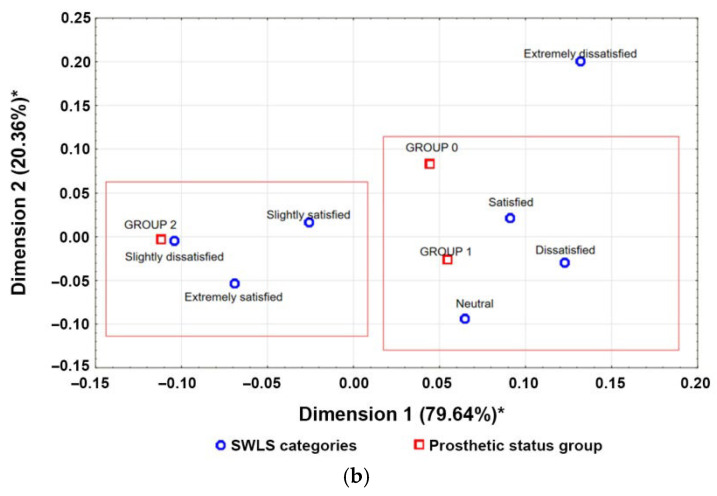
(**a**) Box-and-whisker plot presenting means and standard deviations for the total scores of Satisfaction with Life Scale (SWLS) questionnaire responses across the prosthetic status groups. (**b**) Correspondence map of qualitative life level assessed by Satisfaction with Life Scale (SWLS) questionnaire and prosthetic status groups. Axis 1 and Axis 2 represent the first two dimensions of the correspondence analysis and indicate the variance explained by each dimension.

Mean scores for individual SWLS items across prosthetic groups are shown in Table 4. Cronbach’s α for the scale was 0.85 (all items >0.81). Life satisfaction scores (SWLS) showed highly similar distributions across all three prosthetic groups. Participants generally reported moderately high levels of life satisfaction, with only minimal variation in item-level responses. Figure 2a demonstrates substantial overlap in the interquartile ranges and overall distributions between groups, mirroring the nonsignificant Kruskal–Wallis test (*p* = 0.326). Correspondence analysis showed no clustering of prosthetic groups with specific SWLS satisfaction levels (Figure 2b). Together, these results indicate that prosthetic status was not associated with differences in life satisfaction.

The GOHAI demonstrated acceptable internal consistency (Cronbach’s α = 0.73; all items > 0.65). Mean item responses are shown in Table 5. In contrast to SWLS, the GOHAI revealed clear differences between groups. Participants without dental deficiencies (Group 0) consistently showed more favourable item-level patterns, reporting the highest values (>4.5), except for items on appearance satisfaction (Q7: 3.80), eating without discomfort (Q5: 4.15), and tooth/gum sensitivity (Q12: 4.15).

Individuals with deficiencies—especially RDP users (Group 2)—more frequently reported difficulty eating, dissatisfaction with appearance, or concerns related to teeth or dentures. In Group 1, mean values >4.5 were reported for seven questions, with lower scores for (Q7: 3.15), (Q5: 3.94), (Q12: 4.13), concern over oral health (Q9: 4.14), and biting or chewing (Q2: 4.37). Group 2 showed a similar pattern, with lower scores for Q7 (3.30), Q2 (3.91), Q5 (3.95), Q9 (4.19), and Q12 (4.36).

**Figure 3:**
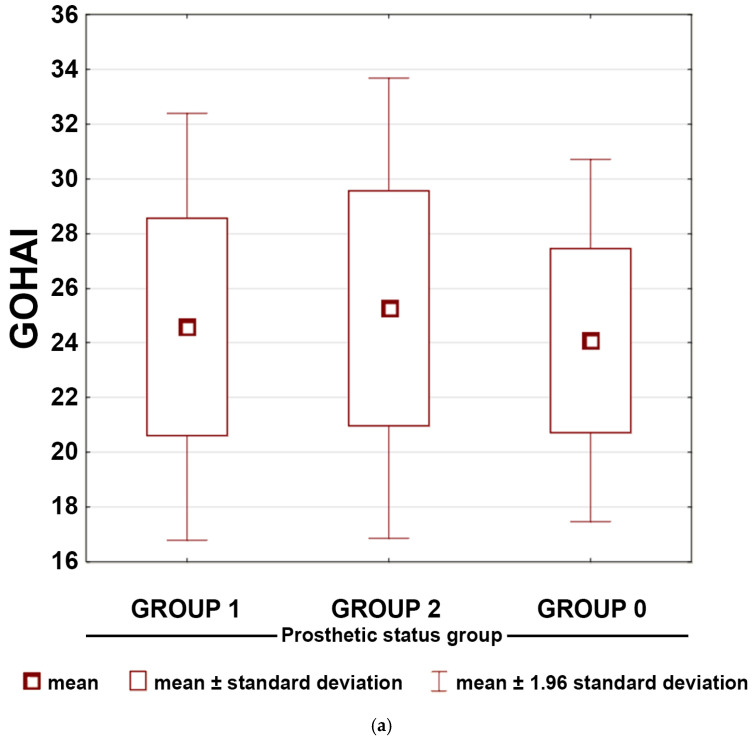

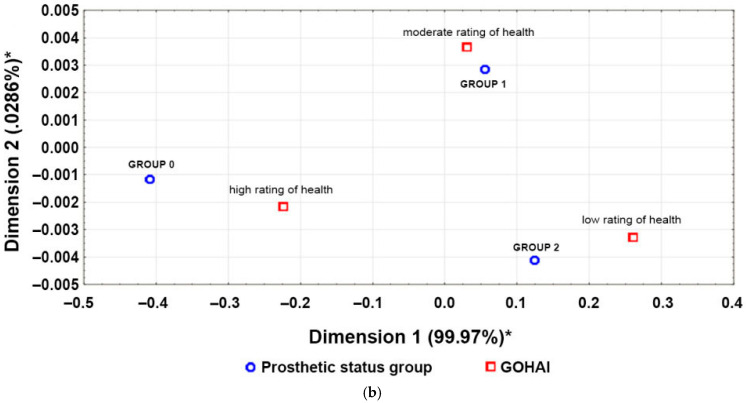
(**a**) Box-and-whisker plot presenting means and standard deviations for the total scores of Geriatric Oral Health Assessment Index (GOHAI) questionnaire responses across the prosthetic status groups. (**b**) Correspondence map of qualitative life level assessed by Satisfaction with Life Scale (SWLS) questionnaire and prosthetic status groups. Axis 1 and Axis 2 represent the first two dimensions of the correspondence analysis and indicate the variance explained by each dimension.

These patterns were reflected in total GOHAI scores: the Kruskal–Wallis test indicated significant variation across groups (*p* = 0.0001). Figure 3a shows that although Groups 1 and 2 partially overlapped, both were shifted toward lower OHRQoL relative to Group 0. Correspondence analysis further demonstrated that Group 0 participants were most closely associated with “very good” OHRQoL classifications, whereas prosthesis users clustered toward “poor” OHRQoL categories (Figure 3b).

## 4. Discussion

### 4.1. Prosthetic Status Patterns in the Study Population

Using data from the Białystok PLUS cohort, we analysed prosthetic status and the use of removable restorations among adults aged ≥50 years. Partial dental deficiency was the most common finding, occurring more often in the mandible than the maxilla. Mandibular defects were also less frequently rehabilitated. Most prostheses were mucosa-supported, reflecting their lower cost and full reimbursement in the Polish public healthcare system [36]. However, such prostheses often provide limited retention and comfort—particularly in the mandible—which may explain their lower uptake. Aesthetic factors may also influence treatment-seeking behaviour, as maxillary defects are more visible than mandibular ones [37]. More advanced restorations, such as tooth- or implant-supported prostheses, remained uncommon, likely due to higher cost and reduced accessibility [38,39].

Socioeconomic factors further shaped prosthetic patterns. In Poland, only mucosa-supported dentures are reimbursed, while all other prosthetic options require private payment [36]. Correspondingly, retirees—who most frequently use reimbursed services—were overrepresented among prosthesis users, whereas unemployed individuals more often lived with unreconstructed deficiencies. Younger, employed participants—who typically have fewer missing teeth—were more often in the group without deficiencies. These findings illustrate how income source, age, and access to services influence prosthetic status.

Complete edentulism was less common than partial loss, yet almost always rehabilitated. Only ~1% of edentulous arches remained unrestored, suggesting that the functional and psychosocial burden of edentulism is high enough that individuals actively seek treatment. This aligns with global evidence identifying edentulism as a major disability worldwide and one of the leading contributors to DALYs [14,18,40].

Sex differences also emerged. Although men typically exhibit more risk factors for tooth loss—such as smoking, alcohol consumption, trauma, and poorer hygiene [36,41,42]—women in our sample were overrepresented among both those without deficiencies and those using prostheses. Men more often had unreconstructed deficiencies, consistent with patterns of lower healthcare utilisation.

### 4.2. Life Satisfaction vs. Oral Health–Related Quality of Life

A key strength of our study was the parallel assessment of life satisfaction and OHRQoL, two related yet distinct constructs. Life satisfaction, measured with the SWLS, remained stable across all groups, averaging 22 points. The broad overlap of SWLS score distributions between groups further supports the finding that global life satisfaction is not meaningfully influenced by prosthetic status in this population. This finding is consistent with evidence showing that life satisfaction is shaped primarily by broader psychological and cultural factors [43] and remains relatively stable in older adulthood, even during major societal stressors such as the COVID-19 pandemic [44,45,46]. The high consistency of SWLS responses across groups reflects this stability, particularly for items known to show strong invariance across populations, such as “In most ways my life is close to my ideal” and “I am satisfied with my life” [47].

In contrast, OHRQoL—measured with the GOHAI—varied significantly between prosthetic groups. Participants without deficiencies reported the highest OHRQoL, whereas prosthesis users reported the lowest, despite rehabilitation. These results mirror findings from other studies in which individuals with unreconstructed partial loss sometimes reported better OHRQoL than prosthesis users [18,38,40]. This pattern suggests that removable prostheses, particularly mucosa-supported dentures, may introduce new sources of discomfort or limitation that offset their intended benefits.

### 4.3. Interpretation of OHRQoL Differences and Functional Dentition

Tooth loss is a recognised contributor to age-related functional decline [2,5,6,8], and OHRQoL has become an important indicator of this aspect of health in older adults. The GOHAI is widely used and considered a standard measure in geriatric oral health research [48]. Our study is one of the few reporting GOHAI findings in Eastern Europe.

Although our analysis focused on total GOHAI scores rather than statistical comparisons of individual items, the descriptive item-level patterns offer additional context. Across all prosthetic groups—including those without dental deficiencies—lower responses were noted for appearance, tooth or gum sensitivity, and eating comfort. These aspects may reflect age-related oral changes or general functional limitations rather than tooth loss alone and warrant further investigation in future studies.

The observation that individuals with untreated partial deficiencies reported better OHRQoL than RDP users suggests that not all missing teeth have equal functional or psychosocial impact. Previous research highlights the importance of the number of occluding tooth pairs, preservation of anterior teeth, and overall occlusal stability [18,38]. Factors such as prosthesis fit, retention, and duration of use—unmeasured in this study—may also significantly influence patient experience [18,49]. These elements should be included in future analyses.

Taken together, our findings underscore that simply counting missing teeth is insufficient to capture the lived impact of oral conditions. They also point to the need to critically reassess traditional hierarchical concepts of functional dentition, which may lead to overtreatment in older adults. Preservation of natural dentition remains strongly associated with higher OHRQoL.

### 4.4. Dietary Limitations and Systemic Health

GOHAI item-level analysis showed that dissatisfaction with appearance and difficulty eating were prominent concerns across groups. Eating comfort remained poor among denture users, consistent with evidence that mucosa-supported prostheses often fail to restore adequate masticatory efficiency [18,49]. Reduced chewing ability has broader implications, as impaired mastication is associated with suboptimal nutrition, increased risk of frailty, metabolic syndrome, diabetes, and cardiovascular disease [12,13]. Recent studies [50,51,52] further emphasise the role of oral function in maintaining metabolic and systemic health in ageing populations. Therefore, reliance on low-cost prostheses may have wider public health consequences beyond oral discomfort alone. Increasing access to tooth- or implant-supported restorations could yield both oral and systemic benefits.

### 4.5. Strengths and Limitations

The main strength of this study lies in its basis within Białystok PLUS—the largest population-based cohort in Poland—providing high-quality, standardised data from a randomly selected urban population. This study used validated questionnaires (SWLS and GOHAI) that had been culturally adapted to Polish conditions and supported by rigorous testing in multiple prior pilot studies [29,30]. The final sample size (n = 986) was three times larger than the minimum required for statistical reliability, reducing the estimation error to 3%. Another advantage is that participants were recruited from the general population rather than from dental clinics, minimising selection bias linked to healthcare use and ensuring inclusion of individuals who may not typically seek dental care.

Despite these strengths, several limitations should be acknowledged. Although this study was based on a population-derived cohort, the response rate was 38%, which may limit representativeness. Non-respondents could differ from participants in socioeconomic status, health behaviour, or oral health awareness, introducing potential selection bias and reducing generalizability. In addition, this study included only urban residents; so, the results cannot be directly extrapolated to rural populations. Comorbidities were not incorporated into the present analysis. Chronic conditions such as diabetes, cardiovascular disease, frailty, multimorbidity, and depression—along with medication use (e.g., xerostomia-inducing drugs)—may significantly influence oral health, prosthetic tolerance, masticatory function, and patient-reported quality of life. Future analyses should account for these factors to better understand causal pathways and interaction effects. Finally, factors such as the number of occluding tooth pairs, prosthesis fit, and duration of prosthesis use were not assessed, though they may significantly influence oral health–related quality of life.

### 4.6. Recommendations for Future Research

Future studies should incorporate more detailed clinical and functional assessments—including the number of occluding tooth pairs, prosthesis fit and retention, duration of prosthesis use, and objective measures of mastication—to better explain variation in OHRQoL. Including comorbidities, medication use, frailty, and nutritional status would further clarify how systemic health interacts with oral function and prosthetic tolerance.

Longitudinal analyses within the Białystok PLUS cohort could help determine temporal relationships between changes in prosthetic status and quality-of-life outcomes. Expanding research to rural and socioeconomically diverse populations, and comparing fixed, tooth- and implant-supported alternatives, would strengthen generalisability.

Clinically, our findings highlight the importance of prioritising preservation of natural dentition and carefully evaluating the expected benefits of mucosa-supported RDPs, which may not enhance—and may reduce—perceived oral health. These insights may guide clinicians toward more individualised and evidence-based prosthetic treatment planning.

## 5. Conclusions

This study demonstrated that prosthetic status is strongly associated with OHRQoL but not with overall life satisfaction. The main findings are:

- Life satisfaction remained stable across all prosthetic groups, indicating that global well-being is not influenced by dental status or prosthesis use.
- OHRQoL differed significantly between groups, with the highest scores among participants without dental deficiencies.
- Users of removable dental prostheses reported the lowest OHRQoL, even lower than those with unreconstructed deficiencies.
- Mucosa-supported prostheses did not improve perceived oral health, suggesting limited effectiveness of this widely used rehabilitation method.
- Preservation of natural dentition is the strongest determinant of favourable OHRQoL, underscoring the importance of preventive and conservative care.

These findings highlight the need to prioritise strategies that maintain natural dentition and to critically evaluate the functional and subjective benefits of removable prosthetic rehabilitation.

## Figures and Tables

**Table 1:** Characteristics of the prosthetic status groups.

Group	Prosthetic Status
0	No dental deficiencies or deficiencies restored with fixed/non-removable prosthetic restorations, i.e., prosthetic crown supported by own tooth or implant, bridge, onlay or overlay restoration
1	Dental deficiencies without prosthetic restoration
2	Dental deficiencies restored with removable prostheses, i.e., full or partial dentures (mucosa-supported), metal framework dentures (tooth support), implant supported dentures

**Table 2:** Demographics of the study population categorized into the prosthetic status groups.

		Prosthetic Status Group		
	Total (n/%)	0—Without Dental Deficiencies (n/%)	1—Dental Deficiencies Not Prosthetically Rehabilitated (n/%)	2—Dental Deficiencies Using Removable Prostheses (n/%)
	986/100	163/16.53	512/51.93	311/31.54
Women	563/57.1	92/56.44	268/52.34	203/65.27
Men	423/42.9	71/43.56	244/47.66	108/34.73
Retired	489/49.59	34/20.86	244/47.66	211/67.84
Pensioner	16/1.62	2/1.23	9/1.76	5/1.61
Employed	433/43.91	115/70.55	233/45.51	85/27.33
Unemployed	48/4.87	12/7.36	26/5.08	10/3.21
Age 50–59	344/34.89	109/66.88	184/35.93	51/16.40
Age 60–69	429/43.51	47/28.83	230/44.92	152/48.87
Age 70–82	213/21.40	7/4.29	98/19.13	108/34.73

**Table 3:** The type and percentage distribution of dental deficiencies and removable prosthetic restorations in the study population.

	Partial Dental Deficiency in the Maxilla (n/% of All Participants)	Partial Dental Deficiency in the Mandible (n/% of All Participants)	Complete Dental Deficiency in the Maxilla (n/% of All Participants)	Complete Dental Deficiency in the Mandible (n/% of All Participants)
Not applicable	416/42.20	325/32.95	853/86.50	903/91.57
Mucosa-supported	211/21.40	145/14.72	116/11.76	67/6.80
Tooth-supported	86/8.71	86/8.72	2/0.20	3/0.30
Implant-supported	2/0.20	2/0.20	1/0.10	1/0.10
Dental deficiencies unrestored	271/27.49	428/43.41	14/1.44	12/1.23

**Table 4:** Satisfaction With Life Scale (SWLS) mean item scores and total score across prosthetic status groups.

	SWLS Questionnaire	Group 0	Group 1	Group 2
	1. In most aspects, my life is close to my ideal.	4.05	4.00	4.01
	2. The conditions of my life are excellent.	4.61	4.59	4.63
	3. I am satisfied with my life.	5.01	5.00	4.89
	4. So far I have gotten the important things I want in life.	4.82	4.72	4.50
	5. If I could live my life over, I would change almost nothing.	4.13	4.17	4.11
Total		22.60	22.45	22.13

**Table 5:** Geriatric Oral Health Assessment Index (GOHAI) mean item scores and total score across prosthetic status groups.

	GOHAI Questionnaire	Group 0	Group 1	Group 2
	1. How often do you limit the kind or amount of food you eat because of problems with your teeth or dentures?	4.86	4.77	4.72
	2. How often do you have trouble biting or chewing any kind of food such as firm meat or apples?	4.77	4.37	3.91
	3. How often are you able to swallow comfortably?	4.93	4.72	4.74
	4. How often do your teeth or dentures prevent you from speaking the way you want?	4.94	4.80	4.74
	5. How often are you able to eat anything without feeling discomfort?	4.15	3.94	3.95
	6. How often do you limit contact with people because of the condition of your teeth or dentures?	4.98	4.91	4.90
	7. How often are you pleased or happy with the appearance of your teeth and gums or dentures?	3.80	3.15	3.30
	8. How often do you use medication to relieve pain or discomfort around your mouth?	4.90	4.85	4.76
	9. How often are you worried or concerned about problems with your teeth, gums or dentures?	4.50	4.14	4.19
	10. How often do you feel nervous or self-conscious because of problems with your teeth, gums or dentures?	4.77	4.53	4.50
	11. How often do you feel uncomfortable eating in front of people because of problems with your teeth or dentures?	4.92	4.73	4.64
	12. How often are your teeth or gums sensitive to hot, cold or sweets?	4.15	4.13	4.36
Total		55.67	53.04	52.71

## Data Availability

The Medical University of Białystok, the implementing body of the Białystok PLUS study under the direction of Professor Karol A. Kamiński, is the legal owner of all used data. Data from the Bialystok PLUS study are available after data application and the signature of a data transfer agreement with the responsible local authorities.

## Abbreviations

The following abbreviations are used in this manuscript:

WHO	World Health Organization
DALYs	Disability-adjusted life years
OHRQoL	Oral health-related quality of life
RDPs	Removable dental prostheses
SWLS	Satisfaction with Life Scale
GOHAI	Geriatric Oral Health Assessment Index
STROBE	Strengthening the Reporting of Observational Studies in Epidemiology
